# Magnetic chitosan/graphene oxide composite loaded with novel photosensitizer for enhanced photodynamic therapy

**DOI:** 10.1039/c8ra00747k

**Published:** 2018-03-14

**Authors:** Xiang Qin, Hongyue Zhang, Zhiqiang Wang, Yingxue Jin

**Affiliations:** Key Laboratory of Photochemistry Biomaterials and Energy Storage Materials of Heilongjiang Province, Harbin Normal University Harbin 150025 China jyxprof@163.com wzq70402@163.com +86-0451-88060569

## Abstract

Photodynamic therapy (PDT) is an increasingly recognized alternative to treat various cancers in clinical practice. Most second-generation photosensitizers (PS) are hydrophobic and have poor targeting selectivity, which limit their efficacy for PDT. In this paper, graphene oxide (GO) coupled with magnetic Fe_3_O_4_ nanoparticles and chitosan (CS) (MCGO) was prepared by a one-pot solvothermal method and used as a nanocarrier for loading the new photosensitizer HNPa (λ_max_ = 698 nm), which was first synthesized by our group, and was considered as a good water-soluble drug and an excellent tissue-penetrating agent due to its strong absorption at 698 nm (near-infrared region). The synthesized composite (MCGO–HNPa) showed high stability, good water solubility and biocompatibility, expected magnetic targetability, and good photostability for PDT even in low concentrations. Our research reveals that MCGO nanomaterials can promote the production and release of singlet oxygen (*Φ*_Δ_ = 62.9%) when compared with free HNPa. In addition, the *in vitro* cell uptake experiments suggested that the MCGO nanomaterials can accelerate the penetration of HNPa drugs into the tumor cell nucleus and that the drug release behavior is pH-sensitive. The MTT assay results against human hepatoma cell lines HepG-2 clearly show that the MCGO–HNPa composite can effectively result in cell damage and apoptotic cell death under light, and that the nanocomposite can improve the PDT antitumor effect of PS agents with negligible dark toxicity. Meanwhile, the research on the photoreaction mechanism reveals that Type I and Type II photodynamic reactions can occur simultaneously in this PDT process, and their relative contributions depend on the type and dose of the photosensitizer. Type II has a greater effect on PDT than Type I, especially for a higher HNPa photosensitizer dose. All the results reveal the promising application of the presented novel strategy.

## Abstract 2

Prepared magnetic chitosan/graphene oxide composite loaded with a novel photosensitizer (MCGO–HNPa) showed excellent PDT activity against HepG-2 cells.

## Graphical abstract

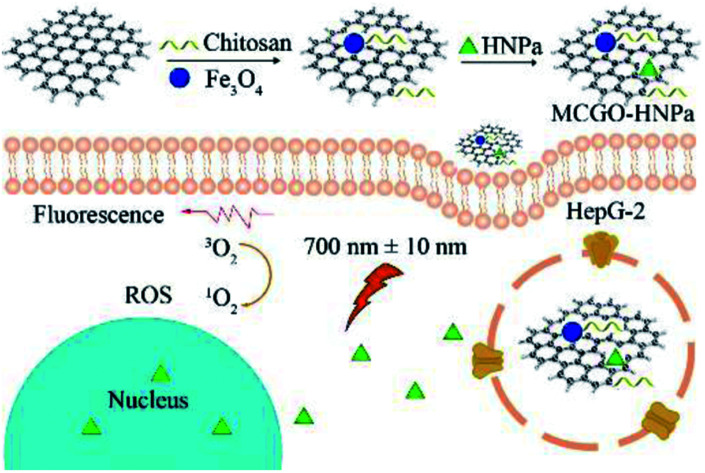

## 1. Introduction

Cancer has become a leading cause of death worldwide and its prevalence continues to increase with increasing population size and urbanization.^1^ Compared with conventional therapeutic modalities such as chemotherapy, surgery, and radiotherapy, photodynamic therapy (PDT) is an increasingly attractive alternative in clinical practice to treat various cancers due to its low side effects, special selectivity and minimally invasive treatment.^2^ It is based on the principle that the interaction between the light of a specific wavelength and the photosensitizer (PS) in tumor tissues generates cytotoxic reactive oxygen species (ROS) including free oxygen radicals and single oxygen created through either electron transfer (Type I) or energy transfer (Type II) photoreactions to inactivate tumor cells.^3,4^

The photosensitizer plays a critical role in PDT. Generally, an ideal PS should have several advantages such as good water solubility, deep penetration in tissues *via* absorption of light of longer wavelengths,^5^ selective accumulation in target tissue, high efficiency in generating ROS within the aerobic tissues,^6^ low dark toxicity, and minimal side effects.^7^ As most second-generation PSs are hydrophobic and have poor solubility in aqueous media; therefore, they cannot be directly injected into the bloodstream.^8^ Through rational structural modification, hydrophilic groups can be introduced to improve water solubility,^9^ or one can increase the penetration depth in PDT by adding conjugate structures to the initial photosensitizer for exerting effective antitumor activity.^10^

Furthermore, to increase the water solubility of PS molecules and improve their delivery into cancer cells, various nanocarrier systems have been designed in recent years.^11,12^ Graphene, which has a large surface area due to its unique two-dimensional (2-D) structure, is a promising drug carrier.^13–15^ Graphene oxide (GO) exhibits favourable biocompatibility, low cytotoxicity and a local thermal effect, which make it a good candidate for a drug-loading/-delivery agent.^16,17^ Functional groups such as epoxy, hydroxyl, and carboxylic acid attached to GO sheets not only enable its good dispersion in physiological environments but also facilitate easy modification with biomolecules, thus improving the stability, water solubility, biocompatibility, or even targetability.^18–20^ Moreover, natural biodegradable polymers are viable polymeric materials that can be used to strengthen the biocompatibility of PS drugs for improving the value of therapeutic molecules.^21^ Among them, chitosan (CS) is an ideal polymer as a nanomaterial modifier for biological applications owing to it being non-toxic, hydrophilic, biocompatible, biodegradable and anti-bacterial.^22–25^ The chelation and cation properties of chitosan promote electrostatic interaction with negatively charged GO sheets. The solubility of chitosan in endosomal pH (5.3) of cancer cells and insolubility in physiological pH (7.4) prevents untimely release of encapsulated drug before reaching the target site.^26^

In addition, nonspecific accumulation in normal tissues of photosensitizer drugs taking a long time can lead to serious side effects and decrease the therapeutic efficacy.^27^ Hence, drug delivery systems need to be primarily directed to tumor sites. Therefore, the development of an efficient delivery system has to focus on the ability to enhance the special target cellular uptake of antitumor drugs, resulting in intelligent and controlled release. Recently, embedding magnetic Fe_3_O_4_ nanoparticles into composites for constructing multifunction materials has been widely reported for targeted drug delivery, with the merits of magnetic separation and magnetic targeting as an external targeting strategy.^28–30^

The assembly of various functional materials on the GO nanosheet surface remains a popular research topic.^31–33^ In this article, through a one-pot solvothermal method, we established a passive targeting system based on magnetic chitosan/graphene oxide (MCGO) composites that contain a biocompatible CS polymer and superparamagnetic iron oxide nanoparticles on the surface of GO, making them promising candidates for magnetic targeting.^34^ Furthermore, this text reports a simple structural modification of a chlorophyll-a degradation product, methyl pyropheophorbide-a (MPPa). A hydrophilic hydroxyl group and a carboxyl group were introduced into MPPa to increase its solubility in the human physiological environment, and the substitution at 13^1^ site ring carbonyl groups with two cyano groups aimed to extend the absorption wavelength of the photosensitizer (*λ*_max_ = 698 nm) to the near-infrared region. The obtained photosensitizer 3-[1-hydroxyethyl]-3-devinyl-13^1^-β,β-dicyanomethylene-13^1^-deoxopyropheophorbide-a (HNPa) was expected to be effective in cancer treatment due to the extended conjugate structure. The novel photosensitizer HNPa as an anti-tumor drug model was then loaded onto the surface of the MCGO nanohybrid *via* π–π stacking and hydrogen bonding.^35^ The composite MCGO–HNPa was comprehensively characterized by various methods. Cellular experiments *in vitro* were conducted to evaluate the potential of the novel photosensitizer HNPa as an anti-tumor drug in PDT and MCGO as an expected magnetic targeting delivery system that can transport anticancer drugs to tumor cells effectively.

## 2. Experimental section

### 2.1. Materials

Methylene cyanide, natural graphite powder, sodium nitrate (NaNO_3_), potassium permanganate (KMnO_4_), hydrogen peroxide solution (H_2_O_2_, 30%), hydrochloric acid, ferric chloride hexahydrate (FeCl_3_·6H_2_O, 98%), sodium acetate (NaOAc), chitosan (CS), dimethyl sulfoxide (DMSO) and 3-(4,5-dimethyl-2-thiazolyl)-2,5-diphenyl-2*H*-tetrazolium bromide (MTT) were purchased from Sigma-Aldrich. 1,3-Diphenylisobenzofuran (DPBF), sodium azide (SA) and d-mannitol (DM) were bought from Sinopharm Chemical Reagent (Shanghai, China). Dulbecco's modified eagle medium (DMEM), penicillin, streptomycin, fetal bovine serum (FBS), and phosphate buffered saline (PBS) were purchased from Beijing Dingguo Biotechnology Co. (Beijing, China). PBS used in other experiments was prepared by mixing stock solutions of NaH_2_PO_4_ and Na_2_HPO_4_. Ethanol, methanol, dichloromethane, triethylamine (TEA), hydrobromide/acetic acid (HBr/HAc, 33%), glacial acetic acid (HAc, 99%), concentrated sulfuric acid (H_2_SO_4_, 98%), and lithium hydroxide (LiOH) were analytical reagents. All the chemicals and reagents were of analytical grade and used without any purification. All the solvents were distilled and purified by standard procedures. Pure water was obtained from Milli-Q synthesis system (Millipore, Billerica, MA, USA).

### 2.2. Chemistry

#### 2.2.1. Synthesis of 3-[1-hydroxyethyl]-3-devinylpyropheo-phorbide-a (HEPa)

125 mg methyl pyropheophorbide-a (MPPa) was added into 10 mL 33% HBr/acetic acid solution with 4 mL 99% glacial acetic acid and stirred at 52 °C for 5 h. The acid and solvent were removed under reduced pressure. The resulting residue was dispersed in 20 mL distilled water and stirred at room temperature for 0.5 h. Then methyl alcohol (20 mL) and 2 M LiOH (5 mL) were added. The solution was refluxed for 2 h in a water bath under nitrogen atmosphere. 2 M acetic acid was added to adjust the pH to 6–7. 60 mL dichloromethane was added to the mixture, and it washed with saturated salt water (120 mL × 3), dried with anhydrous sodium sulfate and filtered. The organic solvent of the filtrate was evaporated in vacuum. The residue was chromatographed using CH_2_Cl_2_/MeOH (25 : 1, v/v) as eluents in 47% (59 mg) yield as a dark blue powder.

#### 2.2.2. Synthesis of 3-[1-hydroxyethyl]-3-devinyl-13^1^-β,β-dicyanomethylene-13^1^-deoxopyropheophorbide-a (HNPa)

100 mg HEPa was dissolved in 4 mL absolute ethanol, and 180 mg methylene cyanide and 195 μL triethylamine were added. The solution was refluxed for 5 h in an oil bath at 85 °C. The mixture was evaporated in vacuum to remove ethanol, the residue was extracted with dichloromethane and washed with saturated salt water several times, and dried with anhydrous sodium sulfate and filtered. The filtrate was evaporated in vacuum. The residue was chromatographed using CH_2_Cl_2_/MeOH (30 : 1, v/v) as eluents in 80% (80 mg) yield as an emerald green powder. ^1^H-NMR (MeOD) *δ* (ppm): 0.01 (brs, 1H, NH), 0.89 (brs, 1H, NH), 1.59 (t, *J* = 7.3 Hz, 3H, 8b-H), 1.74 (d, *J* = 7.3 Hz, 3H, 18-CH_3_), 2.02–2.16, 2.25–2.65 (each m, all 4H, 17a + 17b-H), 2.16 (d, *J* = 6.4 Hz, 3H, 3a-CH_3_), 3.19, 3.21, 3.31 (each s, each 3H, 9H, CH_3_), 3.51–3.64 (q, *J* = 7.3 Hz, 2H, 8a-H), 4.07–4.10 (m, 1H, 17-H), 4.21–4.37 (m, 1H, 18-H), 4.84 (d, *J* = 21.3 Hz, 1H, 13^2^-H), 5.28 (d, *J* = 21.3 Hz, 1H, 13^2^-H), 6.34 (m, *J* = 6.4 Hz, 1H, 3a-H), 8.31 (8.25), 8.46, 9.58 (9.54) (each s, each 1H, 3H, *meso*-H); ^13^C-NMR (MeOD) *δ* (ppm): 9.2, 12.0, 14.4, 17.3, 19.4, 23.7, 30.4, 30.7, 33.0, 48.1, 49.2, 55.1, 94.3, 100.2, 103.5, 105.0, 110.0, 116.0, 115.8, 116.3, 129.9, 131.3, 134.1, 135.3, 137.3, 138.9, 139.1, 144.8, 148.7, 151.8, 158.3, 161.3, 167.1, 173.3, 175.9, 179.5.

#### 2.2.3. Preparation of graphene oxide (GO)

Graphene oxide (GO) was prepared using purified natural graphite according to a modified Hummers method.^36,37^ The typical procedure was as follows: 1 g graphite and 0.5 g NaNO_3_ were added in a 250 mL three-necked round bottom flask, and 23 mL 98% H_2_SO_4_ was added to the mixture under 0 °C using an ice bath and was stirred evenly for 0.5 h below 2 °C. Subsequently, 3 g KMnO4 was added to the reaction system step-wise over 1 h, and the temperature of the mixture was kept below 15 °C. Then, it was stirred for 4 h in an oil bath at 35 °C. Afterwards, 138 mL deionized distilled water was slowly added into the solution, increasing the temperature to 95 °C instantly, while stirring for 0.5 h. Finally, 83 mL deionized distilled water and 67 mL 30% H_2_O_2_ were poured into the reaction system, and bright yellow suspensions were obtained. GO was separated by filtration, and the filter cake was washed with 40 mL 5% HCl and a large amount of deionized distilled water until the filtrate reached a pH = 7, and was dried for 12 h in vacuum. Tan slices of GO were obtained.

#### 2.2.4. Preparation of magnetic chitosan/graphene oxide (MCGO)

0.1 g GO was ultrasonically added to 70 mL ethylene glycol at 70 mL for 1 h using an ultrasonic cell mill to form a clear solution. Then, 0.16 g FeCl_3_·6H_2_O was ultrasonically added to the mixed solution for 15 min. 0.37 g NaOAc was added to the above solution and it was stirred vigorously for 20 min. Subsequently, 0.1 g chitosan already dissolved in 5 mL (2%) acetic acid was added. The mixing solution formed was stirred for 30 min and then placed in a hydrothermal reactor at 185 °C for 6 h. Finally, the solution was cooled to room temperature, and the remaining deposit was washed thoroughly with ethanol and deionized distilled water and dried in a vacuum oven at 50 °C for 12 h.

#### 2.2.5. Material loading photosensitizer (MCGO–HNPa)

The composite was prepared by direct π–π stacking and hydrogen bonding. Typically, magnetic chitosan/graphene oxide was dispersed in absolute ethanol to form a suspension (0.5 mg mL^−1^) by ultrasonic dispersion. 10 mg HNPa dissolved in a small amount of absolute ethanol was cautiously added dropwise. The mixture was ultrasonically treated for 6 h and stirred vigorously for 24 h at room temperature in the dark. The obtained MCGO–HNPa composite nanoparticles were separated by using an external magnet, and washed with absolute ethanol several times to remove unloaded photosensitizer and finally dried at room temperature for 24 h.

### 2.3. Characterization

The microscopic size and shape of the composite were observed when a drop of the sample in absolute ethanol was carefully deposited on carbon-coated copper grids by a Tecnai G2 F200 S-TWIN transmission electron microscope (TEM) (FEI, America) operating at 200 kV. Magnetic characteristics were recorded at 300 K using a 7410 vibrating sample magnetometer (VSM) (Lake Shore, America). Powder X-ray diffraction (XRD) patterns were recorded on a SIEMENS D5005 X-ray diffractometer with a Cu Kα radiation (40 kV, 30 mA) from 10° to 80° and a rate of 3° min^−1^. Raman spectral measurements were carried out using a LabRAM ARAMIS (HORIBA Jobin Yvon). The Fourier transform infrared (FT-IR) spectra were recorded on a Vertex 80 FTIR spectrometer (Bruker Co., Germany) using KBr disc samples in the absorption mode at a resolution of 4 cm^−1^ in the range of 4000–400 cm^−1^. UV-vis spectra were measured using a LAMBDA 25 spectrometer (PerkinElmer) at room temperature in a quartz cuvette with a path length of 1 cm. Fluorescence emission spectra was obtained using a fluorescence spectrometer Fluorolog-3 (Horiba Scientific) with a 150 W xenon lamp as the visible excitation source at an excitation wavelength of 430 nm. The surface charge of the samples in zeta potential and dynamic light scattering (DLS) were carried out using a NanoBrook ZetaPALS zeta potential analyzer (Brookhaven). For the MTT assay, a Biotek ELx800 absorbance microplate reader was used. Cell imaging was performed using a fluorescent inverted microscope (FIM, Leica DM IL IED, Leica Microsystems, Germany).

### 2.4. Drug-loading efficiency and *in vitro* drug release

The amount of HNPa loaded was calculated as follows: first, the standard absorption curve of HNPa was determined by UV-vis spectroscopy at the wavelength of 698 nm from a series of HNPa solutions in different concentrations of ethanol for quantitative analysis. During the MCGO–HNPa fabrication process, the HNPa-containing supernatant was collected and the residual amount of HNPa was determined by measuring the absorbance at 698 nm using UV-vis spectroscopy relative to the above calibration curve recorded under identical conditions, allowing the drug-loading efficiency to be estimated. The drug-loading capacity was calculated according to the following formula:
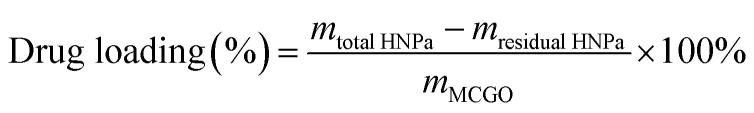
where *m*_total HNPa_ is the total mass of initial drug HNPa for loading, *m*_residual HNPa_ is the mass of residual HNPa in the supernatant after being loaded, and *m*_MCGO_ is the mass of MCGO for loading.

The drug release profile *in vitro* from the synthesized nanocarrier MCGO–HNPa was studied at the physiological temperature of 37 °C and pH of 5.3 (endosomal pH of cancer cells), 7.4 (physiological pH) and 8.9. Briefly, 3 mg of the MCGO–HNPa composite was sealed in a dialysis membrane tube immersed in 10 mL PBS solution with a pH of 5.3, 7.4 or 8.9, followed by placing it in a water bath maintained at 37 °C. An aliquot containing about 2.5 mL drug release medium was withdrawn after 1 h, 3 h, 6 h, and 12 h, and thereafter every 12 h until 72 h. The amount of HNPa in the PBS solution was quantified using UV-vis absorption spectroscopy using the same method as described above. After each measurement, the aliquot was poured back into the release system. Given that the measurement time was very short, while the predetermined time interval of drug release was significantly large, the influence of the returned aliquot on drug release during the measurement time was expected to be insignificant. All the drug release experiments were repeated at least three times.

### 2.5. Singlet oxygen quantum yield

Singlet oxygen production was observed using 1,3-diphenylisobenzofuran (DPBF), a sensitive ^1^O_2_ trapping reagent, in DMF solution. In a typical experiment, 3 mL DPBF (6 × 10^−5^ mol L^−1^) in DMF solution containing 20 μL free HNPa (2.8 × 10^−5^ mol L^−1^) or MCGO–HNPa with the same concentration of the free photosensitizer as the HNPa solutions in DMF was placed in a sealed quartz cuvette. The solution was irradiated by a 10 J cm^−2^ NIR Nd:YAG laser diode at *λ* = 700 nm ± 10 nm every 10 s for 120 s. Each decrease in absorbance caused by photobleaching of DPBF was measured with an ultraviolet-visible spectrophotometer at 415 nm. The ^1^O_2_ quantum yield was calculated using the following equation:

1 *Φ*^S^_Δ_ = *Φ*^R^_Δ_(*κ*^S^*I*^R^_αT_/*κ*^R^*I*^S^_αT_)

2 *I*_αT_ = *I*_0_(1 − e^−2.3*A*_T_^)

where *Φ*_Δ_ is the ^1^O_2_ quantum yield, *I*_αT_ is the total amount of light absorbed by photosensitizers, *A*_T_ is the corresponding absorbance at irradiation wavelength under a specific illumination time *t*, and *κ* is a slope fit by the first-order linear curve plotted against −ln([DPBF]_*t*_/[DPBF]_0_) as a function of irradiation time *t*, where [DPBF]_0_ and [DPBF]_*t*_ represent the UV-vis absorbance of DPBF at 415 nm before and after irradiation time *t*, respectively. S and R represent the sample and reference compound, respectively. The ^1^O_2_ quantum yields of HNPa and MCGO–HNPa in DMF were calculated using methylene blue (*Φ*_Δ_ = 49.1%) as a standard.

### 2.6. Photobleaching of MCGO–HNPa composite

The photostability of the composite was studied as follows: MCGO–HNPa was dissolved in PBS and transferred to a sealed quartz cuvette at three different concentrations of 1.4 × 10^−5^ M, 2.8 × 10^−5^ M, and 5.6 × 10^−5^ M. Then irradiation with a 10 J cm^−2^ NIR Nd:YAG laser diode at *λ* = 700 nm ± 10 nm was performed every 10 min for 60 min. The maximum absorption at 703 nm was recorded using an ultraviolet visible spectrophotometer after irradiation time of 0 min, 10 min, 20 min, 30 min, 40 min, 50 min and 60 min. All experiments were repeated three times and carried out at room temperature and in the dark.

### 2.7. Cell culture

The human hepatocellular carcinoma cell line (HepG-2) was obtained from Harbin Engineering University, Harbin, China. Cells were cultured with Dulbecco's modified Eagle's medium (DMEM, Gibco) containing 10% (v/v) fetal bovine serum (FBS) and 1% antibiotic (100 μg mL^−1^ penicillin–100 μg mL^−1^ streptomycin, Life Technologies, USA) in a 95% humidified atmospheric incubator with 5% CO_2_ at 37 °C.

### 2.8. MTT colorimetric assay

The 3-(4,5-dimethyl-2-thiazolyl)-2,5-diphenyl-2*H*-tetrazolium bromide (MTT) was used to check the cell viability; first, HepG-2 cells were seeded in two 96-well plates at 2 × 10^5^ cells per well in 100 μL DMEM and incubated at 37 °C in 5% CO_2_ for 24 h. Then, 100 μL DMEM containing free HNPa or MCGO–HNPa with a series of equivalent concentrations of HNPa (0.5, 1.5, 2, 2.5, 3, 5 μg mL^−1^) was administered to the cells in the experimental groups and allowed to uptake for 4 h, while the control group was given only 100 μL DMEM without the photosensitizer or the composite. Then cells were fed for 20 h after exposure to calibrated visible light for 10 min (700 nm ± 10 nm, 10 J cm^−2^). Then, MTT solution in DMEM (100 μL, 0.5 mg mL^−1^) was added to each well; after the 4 h incubation with the MTT, the media were removed and 150 μL of dimethyl sulfoxide (DMSO) was added to solubilize the produced formazan crystals. The cell toxicity efficacy was measured with a Biotek ELx800 absorbance microplate reader at a wavelength of 490 nm and then calculated by the following equation:Cell viability (%) = *A*_490(sample)_/*A*_490(control)_ × 100%where *A*_490(sample)_ is the average absorbance values of the wells treated with the same concentration of HNPa or MCGO–HNPa and *A*_490(control)_ is the average absorbance values of the wells treated under the same conditions without the photosensitizer. Data presented are averaged results of six experiments.

### 2.9. Cellular uptake

Cell uptake studies were performed using HepG-2 cells, a human hepatocellular carcinoma cell. To investigate the uptake of MCGO–HNPa composite by HepG-2 cells, the cellular uptake was observed by a Leica DM IL IED fluorescent inverted microscope (FIM) (Wetzlar, Germany). HepG-2 cells were plated into 6-well plates in DMEM at a density of 2 × 10^5^ cells per well and incubated at 37 °C for 24 h. Then the free HNPa or MCGO–HNPa composite was added in the same concentration as the free HNPa photosensitizer (1 mL, 2 μg mL^−1^) to the wells for 0.5 h, 1 h, and 3 h. Afterwards, the cells were washed with 1 mL PBS three times and subsequently fixed with 1 mL of glutaraldehyde aqueous solution (2.5%) for 10 min at 37 °C. Then the glutaraldehyde aqueous solution was removed and the cells were rinsed with PBS three times, and stained with 1 mL of a 1 μg mL^−1^ DAPI nuclear probe for 10 min. Finally, the DAPI dye was removed by rinsing with 1 mL PBS three times, and the cells were placed on a glass slide after removing the coverslip for fluorescence imaging by FIM.

### 2.10. *In vitro* phototoxicity and dark toxicity

The cell culture condition, cell culture medium volume and the cell number used for 96-well plates were similar to that used for the MTT assay. To investigate the cytotoxicity of the MCGO loaded with the anti-tumor drug HNPa towards tumour cells, HepG-2 cells were seeded in two 96-well plates at 2 × 10^5^ cells per well in 100 μL DMEM and incubated at 37 °C in 5% CO_2_ for 24 h. Then 100 μL DMEM containing free HNPa or MCGO–HNPa with a series of equivalent concentrations of HNPa (0.5, 1.5, 2, 2.5, 3, 5 μg mL^−1^) was administered to the cells in the experimental groups and allowed to uptake for 4 h, followed by exposure to calibrated visible light for 10 min (700 nm ± 10 nm, 10 J cm^−2^). After irradiation, the cells were fed for an additional 20 h. In addition, the dark toxicity control group conditions were identical to the experimental group conditions but without irradiation. Analysis of cell cytotoxicity using the MTT assay was conducted as described above.

### 2.11. Morphological changes after PDT

Cell morphological changes after PDT were analysed by DAPI fluorescence staining, which was used to label the nucleus. Typically, HepG-2 cells were seeded in 6-well plates at a density of 2 × 10^5^ cells per well in 100 μL DMEM and incubated at 37 °C in 5% CO_2_ for 24 h. Then the MCGO–HNPa with equivalent concentration of HNPa (1 mL, 2.5 μg mL^−1^) was added for 4 h incubation, and subsequently irradiated for 10 min. Then the cell morphological changes in the bright field were observed by DAPI fluorescence staining after 3 h, 6 h, 9 h, 12 h and 24 h. Briefly, HepG-2 cells were incubated with MCGO–HNPa with an equivalent concentration of HNPa (1 mL, 2.5 μg mL^−1^) and irradiated for 10 min and incubated for 4 h. After removing the culture medium, it was fixed with 1 mL of glutaraldehyde aqueous solution (2.5%) for 10 min, and then 1 mL fluorescence staining DAPI (1 μg mL^−1^) solution was added to the cells in each well covered with a coverslip. Then morphological variation was observed by FIM. The results were compared with those of normal HepG-2 cells.

### 2.12. Type I and Type II reaction mechanism of PDT

During PDT, reactive oxygen species (ROS), such as oxygen centered radicals including hydroxyl radicals (HR), superoxide anions and hydrogen peroxide (Type I reaction mechanism of PDT) and singlet oxygen (Type II reaction mechanism of PDT) play important roles. In order to visualize the photochemical mechanism of PDT, sodium azide (SA), a quenching agent for singlet oxygen, and d-mannitol (DM), an effective scavenger for specific hydroxyl radicals, were used to perform the experiments to quench the ROS generated from a photodynamic reaction. Briefly, the test was divided into four groups: (1) MCGO–HNPa–PDT groups, different concentrations of MCGO–HNPa and irradiation; (2) MCGO–HNPa–PDT–SA groups, different concentrations of MCGO–HNPa with SA (20 μL, 1 mol L^−1^) and irradiation; (3) MCGO–HNPa–PDT–DM groups, different concentrations of MCGO–HNPa with DM (20 μL, 1 mol L^−1^) and irradiation; (4) blank group, without MCGO–HNPa and no irradiation. That is, for the MCGO–HNPa–PDT groups, HepG-2 cells were seeded in 6-well plates at a density of 2 × 10^5^ cells per well in 100 μL DMEM and incubated for 24 h as described above. Then the cells were incubated with different concentrations of MCGO–HNPa for 4 h and irradiated for 10 min. But, for MCGO–HNPa–PDT–SA and MCGO–HNPa–PDT–DM groups, the plate was similarly treated but SA (20 μL, 1 mol L^−1^) and DM (20 μL, 1 mol L^−1^) were added into the culture medium, respectively. The final HNPa equivalent concentrations of MCGO–HNPa were 0.5, 1.5, 2, 2.5, 3 and 5 μg mL^−1^. The cells were further cultured for an additional 24 h as described above. Then DMEM containing MCGO–HNPa was removed and the cells were washed with 1 mL PBS three times. Cell viability was determined by MTT assay.

### 2.13. Statistical analysis

All experiments were performed in triplicate and the data and figures were given as mean ± standard error. Statistical analysis comparisons between two groups were determined by Student's *t*-test using the SPSS 19.0 for Windows (SPSS Inc.). *p* < 0.05 was considered to indicate statistical significance.

## 3. Results and discussion

### 3.1. Chemical synthesis and characterization

**Scheme 1:**
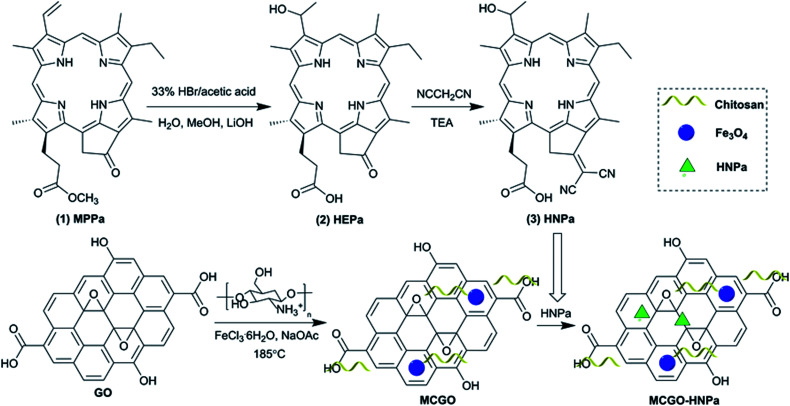
Schematic of the preparation of MCGO–HNPa composite.

The chlorophyll-a based photosensitizer HNPa was prepared as shown in Scheme 1. The first step of the chemical reaction process to obtain the known compound 2 was according to a previously reported procedure.^9^ The second step of the chemical reaction retained the hydrophilic group on the basis of compound 2, and at the same time, the introduction of two strong electron-withdrawing cyano groups in 13^1^-bit extended the conjugated system. The desired product 3-[1-hydroxyethyl]-3-devinyl-13^1^-β,β-dicyanomethylene-13^1^-deoxopyropheophorbide-a (HNPa) (*m*/*z* 600.7094) was obtained in 80% yield. The structure of HNPa was confirmed by ^1^H-NMR spectra, ^13^C-NMR spectra, UV-vis spectra and fluorescence spectra.

The preparation of the magnetic chitosan/graphene oxide composite loaded with the novel photosensitizer HNPa for enhanced photodynamic therapy is also illustrated in Scheme 1. First, graphene oxide (GO) was prepared from purified natural graphite according to a modified Hummers method. Then GO coupled with superparamagnetic Fe_3_O_4_ nanoparticles and biocompatible chitosan (CS) (MCGO) was prepared by a one-pot solvothermal method. The obtained MCGO nanomaterials had high stability, good water solubility and biocompatibility, and expected magnetic targetability for potential use as a drug carrier. Then MCGO was used as a nanocarrier to load the new photosensitizer HNPa (*λ*_max_ = 698 nm) *via* hydrogen bonding interactions and π–π stacking by ultrasound and vigorous stirring.

**Fig. 1:**
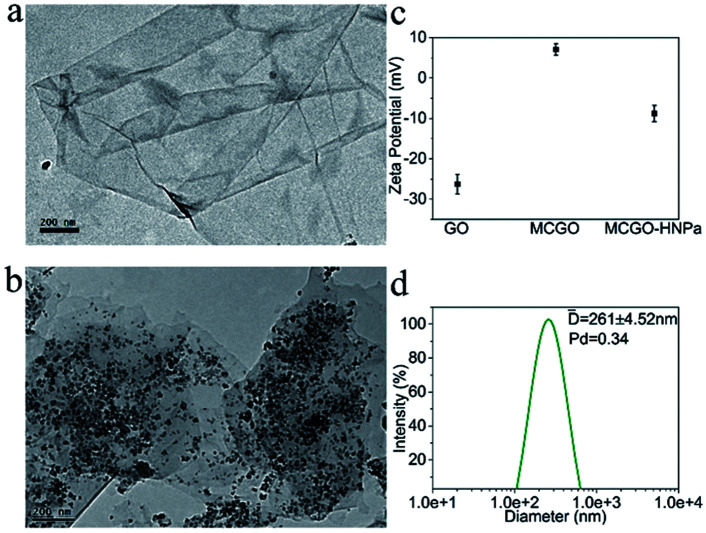
TEM images of GO (a) and magnetic chitosan/graphene oxide composite MCGO (b); (c) zeta potential of GO, MCGO and MCGO–HNPa in ethanol solutions of pH = 7.0. (d) Hydrodynamic size distribution of MCGO–HNPa measured by dynamic light scattering (DLS).

The obtained MCGO–HNPa was characterized by various methods. The morphology of GO in TEM (Fig. 1a) showed a sheet-like structure with a large thickness, smooth surface, and wrinkled edge. Through a one-pot solvothermal method, a passive targeting system was established based on the magnetic chitosan/graphene oxide (MCGO) composite. Then the novel photosensitizer HNPa as an anti-tumor drug model was loaded onto the surface of this nanocomposite. After combination with magnetic nanoparticles, chitosan and HNPa to form the MCGO–HNPa composite, TEM results (Fig. 1b) showed that the magnetic Fe_3_O_4_ spheres were decorated and anchored uniformly in interlayers of GO and many small chitosan molecules and HNPa as the photosensitizer was successfully assembled on the surface of GO layers with a high density.

Zeta potentials of GO, MCGO and MCGO–HNPa in ethanol solutions of pH = 7.0 were further investigated. As shown in Fig. 1c, GO and MCGO nanoparticles showed zeta potentials of −26.28 ± 2.42 mV and 7.08 ± 1.37 mV, respectively, which were attributed to the negative charge of the electronegative groups on the surface of graphene oxide and the positive charge of reactive chitosan–NH_2_, respectively, increasing potential value of GO surface, respectively. After loading with HNPa, MCGO–HNPa showed a zeta potential of −8.81 ± 2.02 mV possibly due to more negative groups of the photosensitizer HNPa. Based on the above investigation, it was clear that HNPa was successfully loaded on the MCGO nanoparticles. Meanwhile, the hydrodynamic diameters of MCGO–HNPa were measured using dynamic light scattering (DLS) shown in Fig. 1d, and the mean diameter of MCGO–HNPa in ethanol was approximately 261 ± 4.52 nm, which was larger than results obtained by TEM. These results could be attributed to the slight aggregation of MCGO–HNPa due to the ultrasonic conditions during the measurement.

**Fig. 2:**
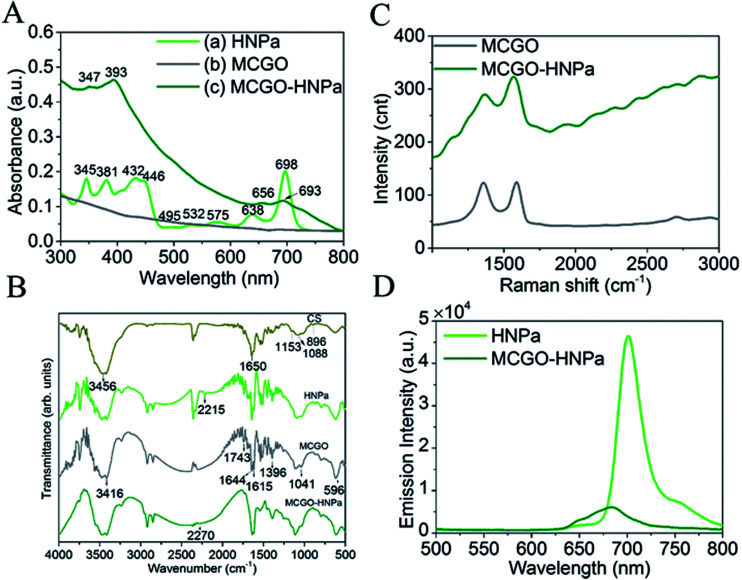
(A) UV-vis spectra of (a) HNPa, (b) MCGO, (c) MCGO–HNPa; (B) FTIR spectra of CS, HNPa MCGO, MCGO–HNPa. (C) Raman spectra of nanomaterial MCGO and MCGO–HNPa composite upon laser excitation at 490 nm. (D) Fluorescence emission spectra of HNPa and MCGO–HNPa. The samples were adjusted to contain the same amount of HNPa and excited at 430 nm.

UV-vis absorption spectra are shown in Fig. 2A. In Fig. 2A(a), HNPa has four intense Soret bands located at 345 nm, 381 nm, 432 nm, and 446 nm and five Q bands at 495 nm, 532 nm, 575 nm, and 638 nm with a low intensity and 698 nm (molar absorption coefficient, *ε* = 29 975 M^−1^ cm^−1^) with a high intensity. An ideal photosensitizer should have near-infrared absorption spectra at long wavelengths, which allows deeper tissue penetration and decreased nonspecific lesions. For instance, 630 nm light penetrates less than 0.5 cm and 700 nm light reaches a depth of no more than 0.8 cm.^38^ Compared with the approved photosensitizer Photofrin (*λ*_max_ = 630 nm, molar absorption coefficient, *ε* = 3000 M^−1^ cm^−1^), HNPa has a stronger absorbance at the longest possible wavelength, which makes it a potential photosensitizer for enhanced PDT.^39^ The MCGO nanohybrid properties before and after loading with HNPa are further confirmed by UV-vis spectra as shown in Fig. 2A(b) and (c). MCGO without HNPa shows virtually no absorption in the range of 300–800 nm. After the MCGO was loaded with HNPa, slight shifts in the UV-vis peaks at around 347 nm, 393 nm, 656 nm and 693 nm attributed to the loaded HNPa molecules are observed in the UV-vis spectrum of MCGO–HNPa. The obvious increase in the baseline of peak pattern is due to the effect of MCGO nanomaterials. The results demonstrate that HNPa molecules are successfully loaded onto MCGO.

The FTIR patterns of CS, HNPa, MCGO and MCGO–HNPa are shown in Fig. 2B. For MCGO, the peaks at 1041, 1396, and 1644 cm^−1^ correspond to C–O–C stretching vibrations, C–OH stretching, and C–C stretching mode of the sp^2^ carbon skeletal network, respectively, while the peaks located at 1743 and 3416 cm^−1^ correspond to C–O stretching vibrations of the –COOH groups, and O–H stretching vibration. There are two characteristic absorbance bands centered at 1615 and 1597 cm^−1^ owing to the C–O stretching vibration of –NHCO– and the N–H bending of –NH_2_, respectively, which proves that –NH_2_ groups on the chitosan chains react with the –COOH groups of GO and therefore are converted to –NHCO– graft points. In addition, the peak at 596 cm^−1^ in the MCGO spectrum is the characteristic Fe–O peak of Fe_3_O_4_. These observations confirm that magnetic Fe_3_O_4_ and CS are successfully grafted on GO. For HNPa, the peak at 2215 cm^−1^ reveals the existence of stretching vibrations of C≡N, while the peak at 2270 cm^−1^ of the MCGO–HNPa is indicative of C≡N stretching vibration of HNPa, which suggests the loading of HNPa on MCGO.

In the fluorescence experiment, the emission spectrum of HNPa shows an intense peak at 703 nm at an excitation wavelength of 430 nm, which is compared with the MCGO–HNPa sample containing the same amount of HNPa. The emission intensity of MCGO–HNPa in Fig. 2D is found to be significantly quenched at the same excitation wavelength as HNPa, suggesting that the energy transfer between HNPa and MCGO is responsible for the low florescence intensity of MCGO–HNPa along with the self-quenching of HNPa.

Raman spectroscopy can be used to characterize the lamellar structure and oxidation degree observed by the ratio of the strength of the D peak to the G peak of graphene oxide.^29^ The Raman spectrum of MCGO in Fig. 2C shows typical Raman spectra of GO, exhibiting peaks corresponding to D, G, and 2D at around 1353, 1583, and 2708 cm^−1^, respectively. After loading with HNPa, the Raman spectra of MCGO–HNPa also shows the characteristic peaks of the GO in MCGO and significantly background upward drift owing to the effect of photosensitizer fluorescence coverage; the decreased intensity in D bands may be due to the ultrasonic stirring reaction process weakening the defect density of MCGO. These results further support the wrapping of HNPa on MCGO.

**Fig. 3:**
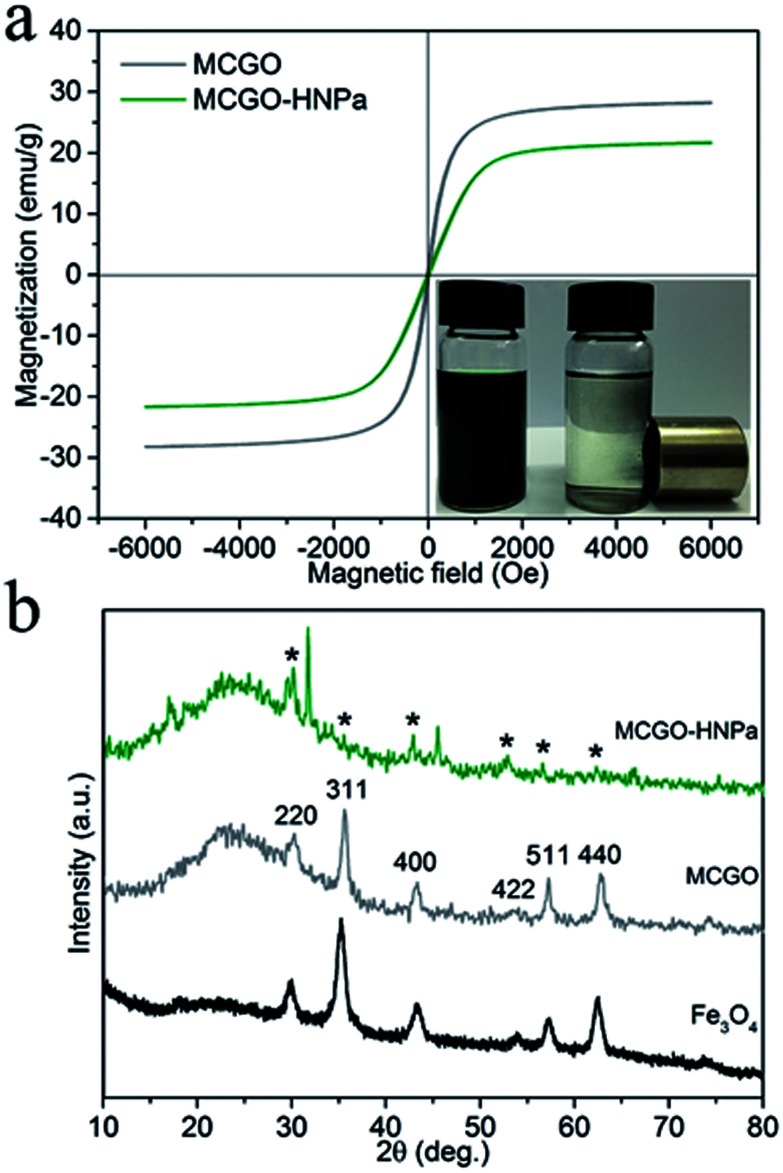
(a) Magnetization loops of MCGO and MCGO–HNPa composite at 300 K. The lower right is the photo of MCGO–HNPa in aqueous solutions without and with a magnet. (b) XRD patterns of pure Fe_3_O_4_, MCGO and MCGO–HNPa, respectively.

Photos of MCGO–HNPa in aqueous solutions without and with a magnet clearly demonstrate its excellent magnetic properties (Fig. 3a). The magnetization hysteresis loop shows that the saturation magnetization of MCGO is about 28.2 emu g^−1^ at 300 K, while that of MCGO–HNPa is about 21.6 emu g^−1^, which further indicates the superparamagnetic nature of MCGO and MCGO–HNPa after drug loading.

XRD patterns of pure Fe_3_O_4_, MCGO and MCGO–HNPa are shown in Fig. 3b. The analysis results are mostly coincident with the six characteristic peaks for Fe_3_O_4_ (2*θ* = 30.1°, 35.5°, 43.1°, 53.4°, 57.1° and 62.6°) observed in the samples, marked by their indices (220, 311, 400, 422, 511, and 440, respectively), indicating the existence of superparamagnetic Fe_3_O_4_ nanoparticles that can be used for the magnetic targeting of PDT. In addition, the XRD patterns of MCGO and MCGO–HNPa show a broad peak from 15° to 30°, which is generally considered to be the diffraction peak of the amorphous structure of chitosan, suggesting that CS is successfully loaded on the surface of MCGO. The decrease in peak intensity of MCGO–HNPa is due to the fact that the content of Fe_3_O_4_ in the nanocomposite is low and that the crystal structure is destroyed after high drug loading. The above analysis clearly shows the superparamagnetic nature of the composite and sheds light on the application of MCGO–HNPa in magnetically targeted PDT.

### 3.2. Drug-loading efficiency and *in vitro* drug release

**Fig. 4:**
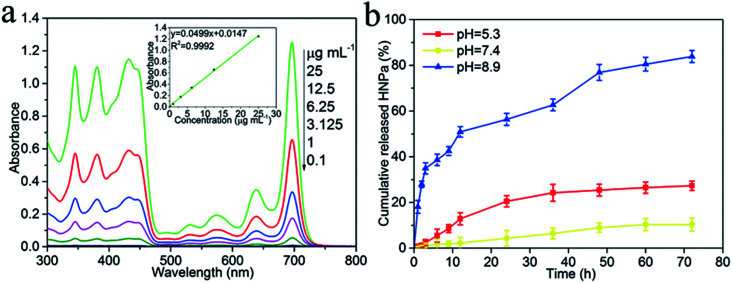
(a) The standard absorption curves of HNPa in ethanol at a wavelength of 698 nm by UV-vis spectra. (b) Percentage of cumulative released HNPa (%) from MCGO drug carrier loading with HNPa (MCGO–HNPa) at 37 °C in phosphate buffer solution (pH = 5.3, 7.4 and 8.9) at different time points. The data points are based on triplicate samples.

HNPa as an anti-tumor photosensitizer drug is loaded on the surface of the multi-functionalized GO (MCGO) *via* a simple sonication mixture method by π–π stacking and hydrophobic interactions. As shown in Fig. 4a, the standard absorption curve of HNPa in ethanol at a wavelength of 698 nm is obtained by linear fitting in the equation *y* = 0.0499*x* + 0.0147 (*R*^2^ = 0.9992). The unbound drug was removed by centrifugation and the loading efficiency of HNPa on MCGO was calculated by measuring the concentration of unbound drugs using UV-vis spectra. The HNPa loading capacities of the MCGO is as high as 57.6% when the initial concentration of the HNPa solution is 1 mg mL^−1^.

The HNPa release from the MCGO–HNPa composite at a temperature of 37 °C in the phosphate buffer solution (pH = 5.3, 7.4 and 8.9) is given in Fig. 4b. The HNPa are released very slowly from multi-functionalized GO (MCGO–HNPa) at neutral conditions, and only about 10.34% of the total bound HNPa is released for 72 h under neutral conditions (pH = 7.4). This is because the hydrogen-bonding interaction between HNPa and the –OH and –COOH groups on MCGO are more prominent at neutral conditions, resulting in an inefficient release. However, in basic conditions, HNPa is released very quickly in the early stage but the release rate gradually declines after 12 h and about 83.88% of the total bound HNPa is released from the nanohybrid in the first 72 h. This may be due to the destruction of the functional groups on the MCGO–HNPa complex under strong alkaline conditions, which destroys the surface structure, resulting in a large amount of HNPa drugs being released. Under acidic conditions, about 27.32% of the total bound HNPa is released for 72 h, which is attributed to the groups of HNPa being protonated, resulting in the partial dissociation of hydrogen bonding. Furthermore, at low pH, the H^+^ in the solution competes with the hydrogen bond-forming group and weakens the above outlined hydrogen-bonding interaction, which may lead to a greater release of HNPa. Hence, the amount of released HNPa from MCGO is much higher under the acidic conditions than under neutral conditions. The interaction between the MCGO sheet and HNPa is due to the π–π stacking as the loading of HNPa on MCGO is still high. In summary, in view of the different releasing behaviors of HNPa on MCGO under different pH environments, this multi-functionalized MCGO can be used as a good candidate material for intelligent drug release.

### 3.3. Singlet oxygen quantum yield

**Fig. 5:**
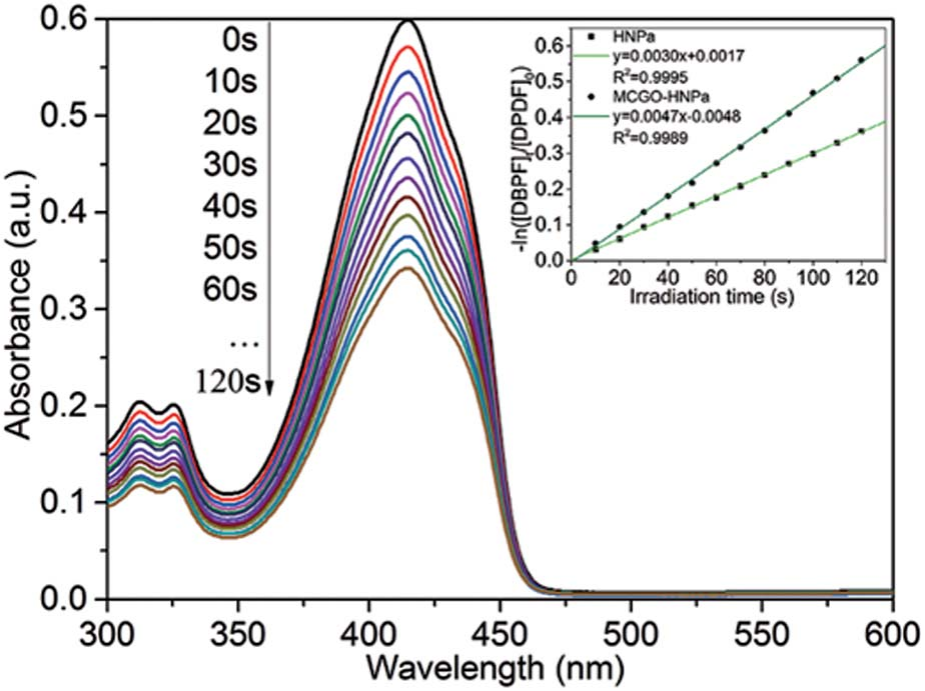
Ultraviolet-visible absorption of DPBF after irradiation of MCGO–HNPa, and first-order plots of the photodecomposition of DPBF photosensitized by HNPa and MCGO–HNPa with an equivalent concentration of HNPa, monitoring the maximum absorption of DPBF at 415 nm.

Singlet oxygen (^1^O_2_) is the vital reactive agent of photosensitizer-induced photodynamic therapy (PDT). Thus, in this experiment, in order to measure the production of ^1^O_2_, 1,3-diphenylisobenzofuran (DPBF), a sensitive ^1^O_2_ trapping reagent, was utilized to check singlet oxygen production in PDT.^9^ The remarkable reduction of DPBF absorbance at 415 nm with increasing irradiation time (700 nm ± 10 nm, 10 J cm^−2^) owing to the quenching reaction with ^1^O_2_ indicated that HNPa could photo-produce ^1^O_2_ efficiently. As shown in Fig. 5, the singlet oxygen quantum yield (*Φ*_Δ_) of HNPa was calculated as 42.6%, while that of MCGO–HNPa with an equivalent concentration of HNPa was 62.9%, using methylene blue as the reference compound (*Φ*_Δ_ = 49.1%). It is clear that MCGO nanomaterials can promote the production and release of singlet oxygen from HNPa to some extent. Compared with HNPa, MCGO–HNPa nanoparticles increased the *Φ*_Δ_ to as high as 62.9%. The result indicated the MCGO–HNPa nanoparticles could enhance photodynamic therapy because the high generation of singlet oxygen would cause excellent cell toxicity, as confirmed by our MTT assay (shown later).

### 3.4. Photobleaching

**Fig. 6:**
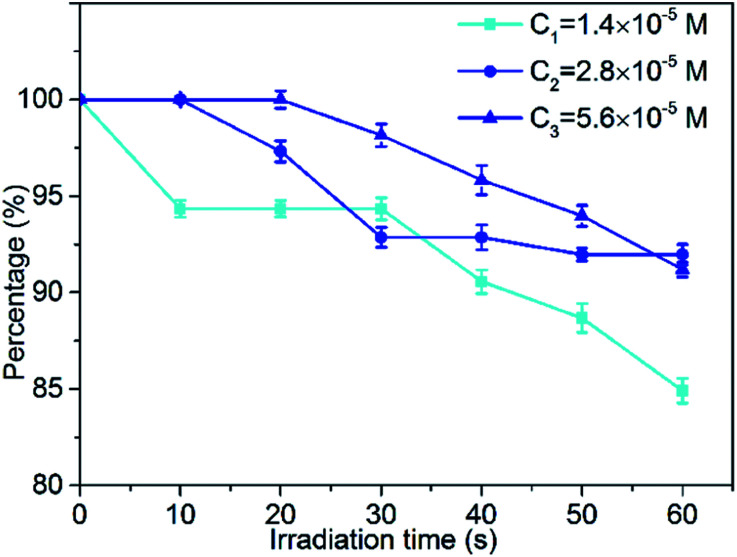
Photobleaching percentage plots of at three different concentrations of (1.4 × 10^−5^ M, 2.8 × 10^−5^ M and 5.6 × 10^−5^ M) MCGO–HNPa composite in PBS with irradiation (700 nm ± 10 nm, 10 J cm^−2^) every 10 min for 60 min.

Photobleaching is the phenomenon accompanying the consumption of photosensitizers during the photodynamic reaction. For the target tissue to be destroyed, photobleaching has a negative effect on reducing the production of reactive oxygen species (ROS) in photodynamic therapy (PDT). The slower the rate of bleaching and the lower the amount of bleaching, the better is the photodynamic therapy. Therefore, optimizing the photobleaching intensity of the photosensitizer is of great significance for the research and application of PDT. In this section, we studied the photobleaching of three different concentrations (1.4 × 10^−5^ M, 2.8 × 10^−5^ M, 5.6 × 10^−5^ M) of the MCGO–HNPa composite in PBS with irradiation (700 nm ± 10 nm, 10 J cm^−2^) every 10 min for 60 min by measuring the UV-vis absorption of the composite at 703 nm after different irradiation times. The photobleaching percentage plots are shown in Fig. 6. After the first irradiation for 10 min, only about 5.7% of the MCGO–HNPa composite was photobleached under the lower concentration of 1.4 × 10^−5^ M. However, under concentrations of 2.8 × 10^−5^ M and 5.6 × 10^−5^ M, the MCGO–HNPa composite was not photobleached at all. After irradiation for 20 min, 5.6 × 10^−5^ M of the MCGO–HNPa composite began to be photobleached. After irradiation for 60 min, 1.4 × 10^−5^ M of the MCGO–HNPa composite was photobleached by about 15%; yet, at concentrations of 2.8 × 10^−5^ M and 5.6 × 10^−5^ M, the photobleaching percentage was about 8% and 8.8%, respectively. This result may be attributed to the concentration of the photosensitizer. According to research, under the same conditions, photobleaching will slow down with an increase in photosensitizer concentration.^40^ All these results indicated that the MCGO–HNPa composite had good photostability for PDT even in lower concentrations.

### 3.5. Cellular uptake

**Fig. 7:**
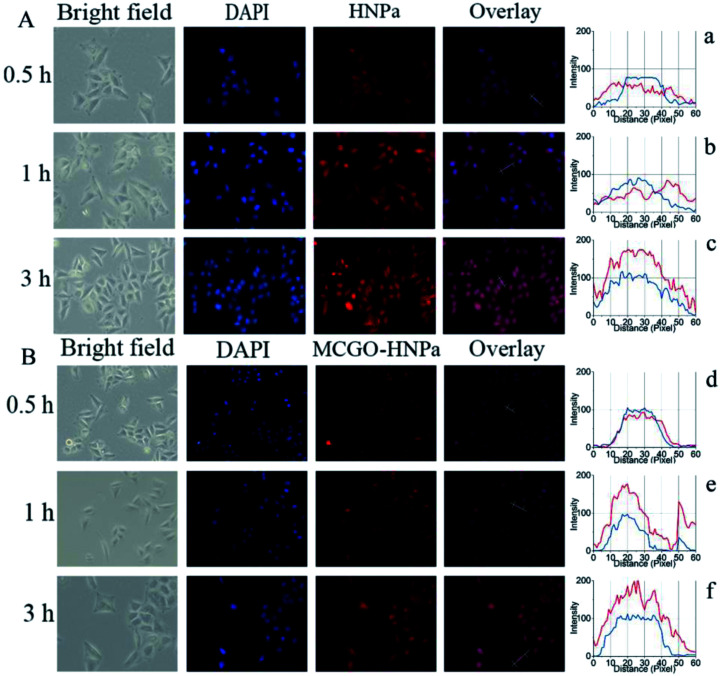
Fluorescence inverted microscopic images of HepG-2 cells after incubation with HNPa (A) or MCGO–HNPa (B) with an equivalent concentration of HNPa (1 mL 2 μg mL^−1^) at 37 °C for 0.5 h, 1 h and 3 h. Blue fluorescence represents the signal of the nucleus stained by DAPI; red fluorescence corresponds to HNPa or MCGO–HNPa. The corresponding cell states are observed in bright field. The fluorescence distribution of cells incubated with HNPa for 0.5 h (a), 1 h (b) and 3 h (c) and with MCGO–HNPa for 0.5 h (d), 1 h (e) and 3 h (f).

The efficient internalization by the cells is crucial for nanohybrids to achieve a better therapeutic efficiency. To examine the cellular delivery of HNPa by the MCGO–HNPa composite in PDT, we compared the cellular uptake behaviors of MCGO–HNPa composite with that of free HNPa. In this study, HepG-2 cells were incubated with free HNPa or MCGO–HNPa with an equivalent concentration of HNPa (1 mL, 2 μg mL^−1^) at 37 °C for 0.5 h, 1 h and 3 h. In order to visualize intracellular uptake, 4′,6-diamidino-2-phenylindole (DAPI), a widely used nuclear staining agent showing blue fluorescence, was utilized to observe the cell nucleus. Cell imaging was performed with a fluorescence inverted microscope. As shown in Fig. 7A, after incubation with HNPa for 0.5 h, the cells showed a very pale red fluorescence in the cytoplasm, indicating that a small amount of the HNPa entered the cells and that there was no obvious change in the nucleus. After incubation for 1 h, the cells showed obviously stronger cytoplasmic red fluorescence inside cells and intensive nuclear red fluorescence indicated that HNPa began to enter the nucleus. After incubation for 3 h, the red fluorescence signal could be detected in almost all cells, particularly in the nucleus, and the fluorescence intensity became stronger than that obtained after incubation for 1 h, which further demonstrated that more and more HNPa entered into the cells with increasing incubation time. These results suggested that HNPa could permeate the tumour cells quickly and effectively. As for the MCGO–HNPa composite shown in Fig. 7B, the process of the cellular uptake behavior of MCGO–HNPa was similar to that of the former free HNPa, but the red fluorescence intensity decreased and the speed of the MCGO–HNPa composite entering the cell nucleus was quicker than that of free HNPa. It could be attributed to the fluorescence quenching of HNPa attached to the MCGO nanomaterials, and the energy retention and conversion of MCGO nanomaterials and released photosensitizer HNPa drugs into cells. All in all, the MCGO–HNPa composite could effectively enter tumor cells with the aim of playing a role in PDT.

Furthermore, the fluorescence distribution images of HepG-2 cells are shown in Fig. 7(a–f). The blue lines represent the fluorescence signal of DAPI, while the red lines represent the fluorescence signal of HNPa. Considering that the DAPI is mainly distributed in the nucleus, it can be seen that the HNPa is mainly located in the cytoplasm in the first 1 h of incubation with HNPa alone. After incubation for 3 h, the HNPa accumulates in the cell nucleus. However, the cells incubated with the MCGO–HNPa composite with increasing time show similar distribution between red fluorescence and blue fluorescence, indicating the HNPa in MCGO–HNPa is mainly located in the cell nucleus. Therefore, the MCGO nanomaterials have an ability to enhance the speed of HNPa drug during entering nucleus.

### 3.6. *In vitro* phototoxicity and dark toxicity

**Fig. 8:**
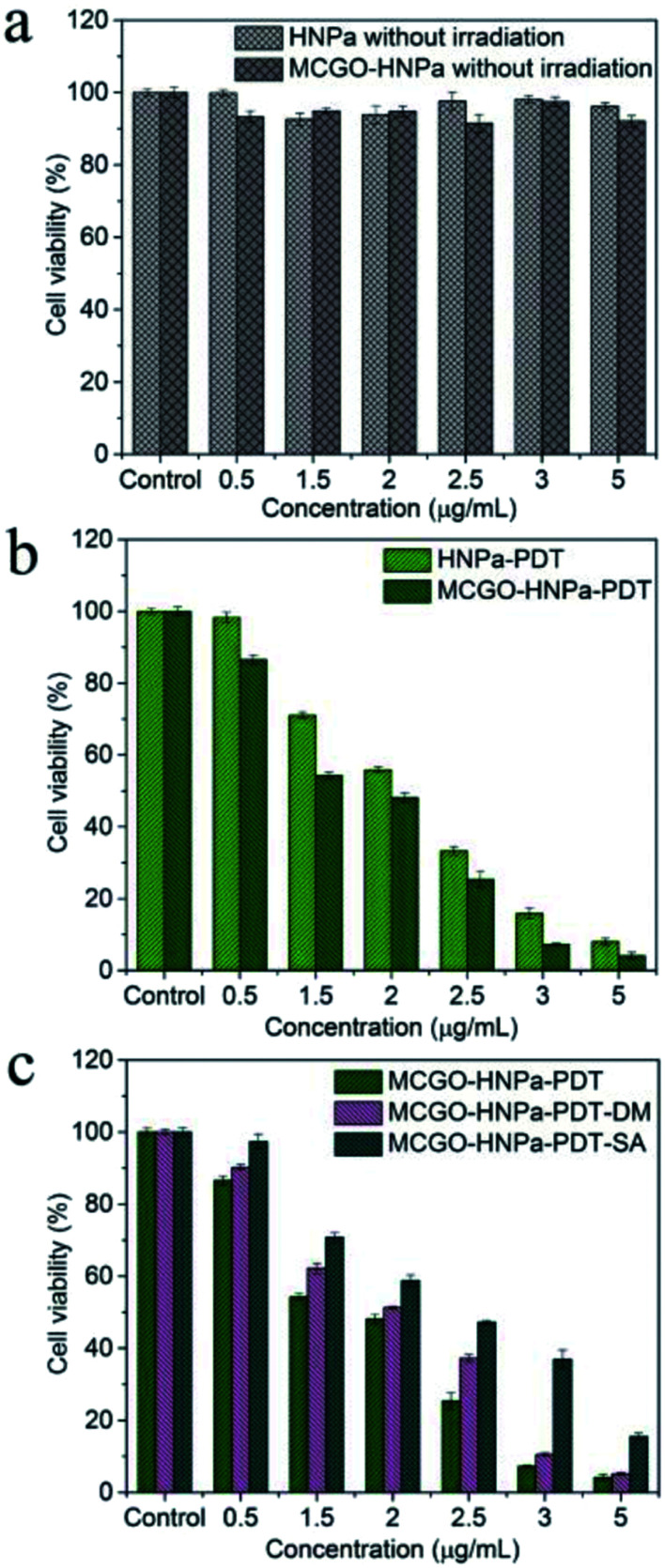
*In vitro* dark toxicity (a) and phototoxicity (b) of HNPa and MCGO–HNPa composite with equivalent concentration of HNPa for HepG-2 cells at different concentrations with irradiation for 10 min. (c) Effect of different reactive oxygen species (Type I and Type II reaction) on HepG-2 cells after PDT for 24 h. MCGO–HNPa–PDT group: different concentrations of MCGO–HNPa (0.5, 1.5, 2, 2.5, 3, 5 μg mL^−1^ of HNPa) and exposed to light. MCGO–HNPa–PDT–DM group: with MCGO–HNPa and DM (a hydroxyl radical quencher) and exposed to light. MCGO–HNPa–PDT–SA group: with MCGO–HNPa and SA (a singlet oxygen quencher) and exposed to light. Cell viability was determined by MTT assay. Data is expressed as mean ± SD (*n* = 3). *P* < 0.05 shows significant difference between groups.

The *in vitro* therapeutic efficacy of PDT using HNPa or the MCGO–HNPa composite was evaluated by MTT colorimetric assay. HepG-2 cells were used for phototoxicity testing, Fig. 8b shows that free HNPa and the MCGO–HNPa composite displayed a concentration-dependent cytotoxicity for cells. HNPa with an increasing concentration ranging from 0.5 to 5 μg mL^−1^ with irradiation time of 10 min exhibited a significant gradual reduction in cell viability. HNPa showed a cell viability of 98.32% at 0.5 μg mL^−1^ after PDT, while at 5 μg mL^−1^, the cell viability was 8.01% under the same light and culturing conditions. When incubated with the MCGO–HNPa composite, the cell viability decreased to 86.52% and 4.11% after PDT for composite concentrations of 0.5 μg mL^−1^ and 5 μg mL^−1^, respectively. Moreover, the IC_50_ value (1.504 ± 0.522 μg mL^−1^) of the MCGO–HNPa composite was lower than that of free HNPa (2.013 ± 0.138 μg mL^−1^) by exposure to calibrated visible light for 10 min (700 nm ± 10 nm, 10 J cm^−2^), suggesting that the MCGO–HNPa composite could enhance photodynamic therapy efficiency to a certain extent, which may be attributed to the higher ^1^O_2_ quantum yield of the MCGO–HNPa composite reported previously. Meanwhile, as shown in Fig. 8a, the average cell viability of the dark control group was more than 90% in a dose-dependent manner, which indicated that the cell death was only induced by a combination of photosensitizer and light. Although the MCGO–HNPa composite showed a slightly lower cell viability than HNPa under identical conditions, it still exhibited low relative dark toxicity. The results clearly suggested that the MCGO–HNPa composite was able to enhance the effect of PDT agents for improved photodynamic cancer cell killing.

### 3.7. Morphological changes after PDT

In order to study the specific process of photosensitizer action on cells after PDT, the cell morphological changes of HepG-2 cells in the bright field were analyzed by a fluorescent inverted microscope (FIM). The control group (0 h) cells without treatment with the MCGO–HNPa composite presented a spindle-shaped normal cell morphology. However, it was clear that the HepG-2 cells incubated with the MCGO–HNPa composite (2.5 μg mL^−1^) after PDT for 3 h, 6 h, 9 h, 12 h and 24 h gradually became rounder and their shading degree decreased significantly. It showed that the tumor cells were damaged due to necrosis and apoptosis with increasing PDT time. After PDT for 3 h, the cells began to become brighter and rounder. After PDT for 12 h, most of the cells were destroyed and died. After PDT for 24 h, almost all the cells were dead, and the number of cells in the field of vision reduced because of the decreased cell adhesive ability, resulting in partially dead cells detached from the wall of the culture dish. Based on the above results, the morphological variation of cells clearly indicated that the MCGO–HNPa composite under light could effectively result in cell damage and apoptotic cell death in HepG-2 cells, which was in good agreement with results of the photodynamic activity of MCGO–HNPa.

### 3.8. Type I and Type II mechanism of PDT

PDT involves the use of a three-component system comprising a photosensitizer, light of specific wavelength and molecular oxygen, resulting in the formation of reactive oxygen species (ROS) including singlet oxygen and free oxygen radicals that are directly responsible for the death of cancer cells. The reaction mechanism can be of two types. Photoactive drug photosensitizer, which is administered to cancer cells followed by appropriate wavelength light activation, can be excited form the ground state to the excited triplet state T to produce free oxygen radicals (type I photoreactions) such as hydroxyl radicals (HR), superoxide anions and hydrogen peroxide through electron transfer from a substrate molecule or singlet oxygen (^1^O_2_) (Type II photoreactions) to tissue oxygen through energy transfers. These ROS show very strong activity to induce cell death and necrosis of tumor components. In order to visualize the photochemical processes mechanism of PDT, corresponding ROS of Type I and Type II generated from the photodynamic reaction were quenched using specific quenching agent DM (a hydroxyl radical quencher) and SA (a singlet oxygen quencher), respectively. As shown in Fig. 8c, the cell viability of the MCGO–HNPa–PDT–DM group and MCGO–HNPa–PDT–SA group were obviously higher than that of the MCGO–HNPa–PDT group, indicating that the ROS of the hydroxyl radical and ^1^O_2_ generated from the MCGO–HNPa composite in the HepG-2 cells after PDT has been quenched effectively. In addition, the cell viability of the MCGO–HNPa–PDT–SA group was much higher than that of the MCGO–HNPa–PDT–DM group with increasing concentration of HNPa, suggesting that the production of ^1^O_2_ is more than that of the hydroxyl radical. In conclusion, Type I and Type II photodynamic reactions can occur simultaneously in this PDT, and their relative contributions depend on the type and dose of photosensitizer. Type II has a greater effect on PDT than Type I, especially in a higher photosensitizer dose.

## 4. Conclusions

In summary, the magnetic Fe_3_O_4_ spheres were decorated and anchored uniformly in interlayers of GO and many small chitosan and photosensitizer HNPa molecules were successfully assembled on the surface of the GO layers with a high density. The *in vitro* drug release, ^1^O_2_ production, photobleaching, cell uptake, *in vitro* phototoxicity and dark toxicity, cytomorphology, and photoreaction mechanism of the MCGO–HNPa composite were comprehensively investigated. The MCGO–HNPa exhibited no obvious dark toxicity and good photostability. Compared with the free HNPa under the same experimental conditions, the MCGO–HNPa composite showed a higher singlet oxygen quantum yield (*Φ*_Δ_) (62.9%) and efficient cell toxicity against human hepatocellular carcinoma cell line (HepG-2) (IC_50_ = 1.504 ± 0.522 μg mL^−1^) by irradiation for 10 min (700 nm ± 10 nm, 10 J cm^−2^). Furthermore, cellular uptake experiments suggested that the MCGO nanoparticles could accelerate the penetration of HNPa drugs into tumor cell nuclei. In addition, the photoreaction mechanism investigations showed that Type I and Type II photodynamic reactions could occur simultaneously in this PDT and Type II had a greater effect on PDT than Type I, especially in a higher photosensitizer dose. All the results showed that the prepared MCGO–HNPa composite had great potential application for PDT.

## Conflicts of interest

There are no conflicts to declare.

## Supplementary Material
